# Differences in expression of the cancer stem cell marker aldehyde dehydrogenase 1 among estrogen receptor-positive/human epidermal growth factor receptor type 2-negative breast cancer cases with early, late, and no recurrence

**DOI:** 10.1186/s13058-016-0731-3

**Published:** 2016-07-02

**Authors:** Yuichiro Miyoshi, Tadahiko Shien, Akiko Ogiya, Naoko Ishida, Kieko Yamazaki, Rie Horii, Yoshiya Horimoto, Norikazu Masuda, Hiroyuki Yasojima, Touko Inao, Tomofumi Osako, Masato Takahashi, Nobumoto Tomioka, Yumi Endo, Mitsuchika Hosoda, Hiroyoshi Doihara, Shinichiro Miyoshi, Hiroko Yamashita, Collaborative Study Group of Scientific Research of the Japanese Breast Cancer Society

**Affiliations:** Department of General Thoracic Surgery and Breast and Endocrine Surgery, Okayama University Graduate School of Medicine, Dentistry, and Pharmaceutical Sciences, 2-5-1 Shikata-cho, Okayama-city, Okayama 700-8558 Japan; Breast Surgical Oncology, Cancer Institute Hospital, Japanese Foundation for Cancer Research, 3-8-31 Ariake, Koto-ku, Tokyo, 135-8550 Japan; Breast Surgery, Hokkaido University Hospital, Kita-14, Nishi-5, Kita-ku, Sapporo, Hokkaido 060-8648 Japan; Division of Pathology, Cancer Institute Hospital, Japanese Foundation for Cancer Research, 3-8-31 Ariake, Koto-ku, Tokyo, 135-8550 Japan; Department of Breast Oncology, Juntendo University School of Medicine, 3-1-3 Hongo, Bunkyo-ku, Tokyo, 113-8431 Japan; Breast Oncology, Department of Surgery, National Hospital Organization (NHO) Osaka National Hospital, 2-1-14 Hoenzaka, Chuo-ku, Osaka, Osaka 540-0006 Japan; Department of Breast and Endocrine Surgery, Graduate School of Medical Science, Kumamoto University, 1-1-1 Honjo, Chuo-ku, Kumamoto, Kumamoto 860-8556 Japan; Department of Breast and Endocrine Surgery, Kumamoto City Hospital, 1-1-60 Kotoh, Higashi-ku, Kumamoto, Kumamoto 862-8505 Japan; Department of Breast Surgery, National Hospital Organization (NHO) Hokkaido Cancer Center, 4-2-3-54 Kikusui, Shiroishi-ku, Sapporo, Hokkaido 003-0804 Japan; Department of Oncology, Immunology and Surgery, Nagoya City University Graduate School of Medical Sciences, 1 Kawasumi, Mizuho-cho, Mizuho-ku, Nagoya, 467-8601 Japan; Present address: Kumamoto Shinto General Hospital, 1-17-27 Shinyashiki, Chuo-ku, Kumamoto, Kumamoto 862-8655 Japan

**Keywords:** Breast cancer, Estrogen receptor-positive, Human epidermal growth factor receptor type 2-negative, Aldehyde dehydrogenase 1, Time of recurrence

## Abstract

**Background:**

The significance of the expression of aldehyde dehydrogenase 1 (ALDH1), a cancer stem cell marker, for predicting the recurrence of estrogen receptor (ER)-positive/human epidermal growth factor receptor type 2 (HER2)-negative breast cancer is still poorly understood. The value of ALDH1 in predicting the time of recurrence remains unknown.

**Methods:**

In total, 184 patients with early distant recurrence, 134 patients with late distant recurrence, and 321 control patients without recurrence for more than 10 years after starting initial treatment for ER-positive/HER2-negative breast cancer, registered in 9 institutions, were analyzed. We assessed relationships between ALDH1 and other clinicopathological features, and ALDH1 expression was compared among the three groups. The relationship between ALDH1 expression and overall survival after recurrence was also evaluated in each group.

**Results:**

The rates of ALDH1 expression positivity (more than 1 %) in the early, late, and no recurrence groups were 18.4 %, 13.4 %, and 8.4 %, respectively. ALDH1 expression correlated significantly with lymph node metastases (*p* = 0.048) and the Ki-67 labeling index (*p* < 0.001) in the early recurrence group. Multivariate analysis revealed ALDH1 expression to be significantly higher in the early recurrence group than in the no recurrence group (adjusted OR 2.140, 95 % CI 1.144–4.003, *p* = 0.016). Moreover, there was a significant difference in ALDH1 expression between the early and no recurrence groups receiving adjuvant endocrine therapy and chemotherapy (adjusted OR 4.625, 95 % CI 1.881–12.474, *p* < 0.001). However, there was no difference in ALDH1 expression between the late and no recurrence groups in univariate analysis (OR 1.507, 95 % CI 0.738–2.998, *p* = 0.253). In multivariate analysis, ALDH1 was not a factor independently predicting overall survival after the detection of recurrence (adjusted OR 1.451, 95 % CI 0.985–2.085, *p* = 0.059).

**Conclusions:**

Among patients with ER-positive/HER2-negative breast cancer, ALDH1 expression was more common in those with early recurrence, and this expression was found to be associated with a more aggressive breast cancer phenotype than that in the patients without recurrence. Further study is needed to clarify the prognostic significance of the heterogeneity of cancer stem cells and to confirm their role in resistance to chemotherapy.

## Background

For patients with estrogen receptor (ER)-positive/human epidermal growth factor receptor 2 (HER2)-negative breast cancer receiving adjuvant chemotherapy, endocrine therapy for 5 years plus additional chemotherapy for high-risk groups, such as patients with high tumor burden, advanced histological grade, and/or strong Ki-67 expression, is considered to improve survival. This combined treatment strategy was proven to be effective in reducing the risk of recurrence within the first 5 years after diagnosis [1]. However, some tumors give rise to recurrence regardless of adjuvant therapy, and there are reports indicating the efficacy of chemotherapy to be less for patients with ER-positive breast cancer than for those with ER-negative breast cancer [2]. Furthermore, women with ER-positive breast cancer remain at particularly high risk of late recurrence, which is defined as relapse more than 5 years after initial treatment, and prolongation of endocrine treatment duration is thus under discussion [3]. Therefore, accurate and reliable estimates of the risk of recurrence after 5 years of endocrine therapy are necessary for making the most appropriate decisions regarding extended endocrine therapy. Tumor size and nodal metastases were previously reported to be predictive factors for early and late recurrence of ER-positive/HER2-negative tumors [4, 5]. The gene signature assay, homeobox/interleukin-7 ratio, Breast Cancer Index (Biotheranostics, San Diego, CA, USA), Prosigna assay (NanoString Technologies, Seattle, WA, USA), Oncotype DX assay (Genomic Health, Redwood City, CA, USA), and EndoPredict test (Sividon Diagnostics, Cologne, Germany) have all been employed in efforts to predict early and late recurrence events in patients with breast cancer. Risk factors reliably associated with not only early but also late recurrence need to be determined, and those patients who would benefit from extended endocrine therapy must be identified.

Recently, aldehyde dehydrogenase 1 (ALDH1) was recognized as a cancer stem cell marker [6]. ALDH1 is a detoxifying enzyme that oxidizes aldehyde and thereby impacts resistance to alkylating agents [7]. ALDH1 also converts retinol into retinoic acid. Retinoic acid, which is a modulator of cell proliferation, might influence the proliferation of cancer stem cells [8]. Therefore, ALDH1-positive cells may contribute to the development of resistance to adjuvant chemotherapeutic agents and the aggressiveness of malignant tumors [9–12]. In a clinical study, Ginestier et al. initially found ALDH1-positive tumor cells to be associated with poor clinical outcomes [6]. Morimoto et al.’s report also showed ALDH1-positive breast cancers to have an aggressive phenotype [13]. Dong et al. reported ALDH1 expression to be an independent predictor of poor outcomes in patients with breast cancer [14]. However, in other previous studies, researchers found ALDH1 expression to be associated with good outcomes. Thus, the significance of ALDH1 expression as a predictor of the recurrence period for ER-positive/HER2-negative breast cancer has yet to be elucidated.

Previously, we investigated clinicopathological factors predictive of early and late recurrence in ER-positive/HER2-negative breast cancer cases in a joint multi-institutional study carried out by the Collaborative Study Group of the Scientific Research of the Japanese Breast Cancer Society. The results of that study suggest that predictors of early and late distant recurrence may differ according to menopausal status and age [15]. We next explored a new biomarker for predicting the recurrence period in the same cohort. We noted that cancer stem cells could remain in a dormant state and thereby escape the effects of adjuvant therapy. Such cells might grow relatively slowly for a long period and eventually become detectable metastatic tumors. We hypothesized that ALDH1 expression in the primary lesion correlates with early and late recurrence of ER-positive/HER2-negative breast tumors. We retrospectively collected data from ER-positive/HER2-negative breast cancer cases with early and late distant recurrence and from patients who remained recurrence-free for more than 10 years. We compared ALDH1 expression levels among these three groups. The relationship between ALDH1 expression and overall survival after recurrence was also evaluated.

## Methods

### Cases and clinical samples

In total, 223 consecutive patients with early distant recurrence and 149 consecutive patients with late distant recurrence of ER-positive/HER2-negative breast cancer, who had undergone breast surgery and/or neoadjuvant chemotherapy between January 2000 and December 2004, were registered from 9 institutes. These institutes were Okayama University, Cancer Institute Hospital, Japanese Foundation for Cancer Research, the Hokkaido University Institutional Review Board (IRB), Juntendo University, National Health Organization (NHO) Osaka National Hospital, Kumamoto University, Kumamoto City Hospital, NHO Hokkaido Cancer Center, and Nagoya City University. This was a joint multi-institutional study titled Analysis of Biological Characteristics and Factors Predicting Late Recurrence in Breast Cancer, carried out by the Collaborative Study Group of Scientific Research of the Japanese Breast Cancer Society. Early recurrence was defined as being within 5 years of initial treatment, late recurrence as more than 5 years after initial treatment. For each late recurrence patient, approximately two age-matched patients free of recurrence for more than 10 years were randomly selected using the RAND function in Excel software (Microsoft, Redmond, WA, USA) at each institution. In total, 321 patients who had been recurrence-free for more than 10 years served as study controls. The study protocol was approved by the IRB of each participating institution and conformed to the guidelines of the 1996 Declaration of Helsinki. Informed consent was obtained from some of the patients. However, opting out and a waiver of informed consent were options, as anonymized archival specimens were used in this retrospective study.

Among the registered patients, formalin-fixed, paraffin-embedded samples (*n* = 184 early recurrence, *n* = 134 late recurrence, *n* = 321 no recurrence) were available from 639 individuals. ALDH1 expression rates were compared among the three groups. We also assessed the relationships between ALDH1 and other clinicopathological features. Moreover, the relationship between ALDH1 expression and overall survival after recurrence was evaluated for each of the groups.

### Immunohistochemical staining and scoring for ER, progesterone receptor, HER2, and Ki-67 expression

Expression of ER, progesterone receptor (PgR), HER2, and Ki-67 were centrally assessed with immunohistochemical (IHC) staining techniques. IHC staining was performed on formalin-fixed, paraffin-embedded sections (3–4 μm). The primary antibodies used included monoclonal mouse antihuman ERα antibody (1D5; Dako, Glostrup, Denmark) at a 1:100 dilution for ER, monoclonal mouse antihuman PgR antibody (636; Dako) at a 1:100 dilution for PgR, and monoclonal mouse antihuman Ki-67 antibody (MIB-1; Dako) at a 1:200 dilution. ER was considered to be positive if nuclear staining exceeded 1 %. The Ki-67 labeling index was assessed as the percentage of tumor cells showing definite nuclear staining among more than 1000 invasive tumor cells. HER2 immunostaining was evaluated using the HercepTest (Dako). Tumors with a score of 2+ were tested for gene amplification by in situ hybridization (ISH). Tumors were considered to be HER2-positive if IHC staining was scored 3+ or if ISH results were positive. ER-negative and/or HER2-positive tumors were excluded from this study.

### IHC staining and scoring for ALDH1

IHC staining of formalin-fixed, paraffin-embedded sections (thickness:3–4 μm) was performed. Mouse anti-ALDH1 antibody (aa 7–128, catalog number 611195; BD Biosciences, San Jose, CA, USA) was diluted 1:1000. An immunohistochemistry kit containing horse serum blocking liquid and secondary antibody (catalog number MP-7402; Vector Laboratories, Burlingame, CA, USA) and A 3,3′-diaminobenzidine (DAB) kit (catalog number SK-4105; Vector Laboratories) were used. Antigen retrieval was performed in citrate buffer according to the manufacturer’s protocol. The diluted ALDH1 antibody was added to the available specimens and left standing for 1 h at room temperature. PBS, instead of the first antibody, was added to the specimens used as negative controls. Liver tissue specimens were also used as known positive controls for IHC staining. Secondary antibody and HRP-labeled streptavidin were added to the breast cancer specimens, followed by DAB color rendering and counterstaining with hematoxylin. ALDH1 staining of tumor cells was considered to be positive when the cytoplasmic cellular components showed a positive reaction. Special attention was paid to stromal cells, lymphocytes, and histiocytes because such cells show cross-reactions with ALDH1. Therefore, hematoxylin and eosin-stained sections were also used to distinguish ALDH1-positive tumor cells from other stained cells. One investigator scored ALDH1 expression rates. However, unclear findings, especially when it was difficult to distinguish ALDH1-positive tumor cells from other positive cells, were encountered. Such findings were discussed with both another investigator and one pathologist, employing a multihead microscope. Differences of opinion were resolved by discussion until consensus was reached.

### Statistical analysis

Differences in clinicopathological data were compared with the chi-square test. In the comparison between cases with recurrent disease and the recurrence-free patients, the ORs for different variables were assessed by applying a logistic regression model in univariate and multivariate analyses. The Kaplan-Meier method was used to estimate overall survival from the time of recurrence. Differences between overall survival curves were determined with a log-rank test. For both univariate and multivariate analyses, Cox regression was used to evaluate the influence of the variables on survival. All of the data were analyzed with the use of JMP 11.0.0 statistical software (SAS Institute, Cary, NC, USA). *p* < 0.05 was considered to indicate a statistically significant difference.

## Results

### Patient characteristics

Patient characteristics are presented in Table 1. The median follow-up durations were 72 (range 14–179) months, 133 (range 67–177) months, and 128 (range 57–179) months in the early, late, and no recurrence groups, respectively. During follow-up of these 639 patients, 69.5 % (128 of 184) of those with early recurrence and 31.3 % (42 of 134) of those with late recurrence died as a result of breast cancer. The histology was invasive ductal carcinoma in 94.0 % (173 of 184), 93.2 % (125 of 134), and 93.1 % (299 of 321) of the early, late, and no recurrence groups, respectively. The recurrences were local in 20.1 % (37 of 184) of the early recurrence group and in 28.3 % (38 of 134) of the late recurrence group. Adjuvant endocrine therapy alone had been administered to 28.8 % (53 of 184) of early recurrence patients, 41.0 % (55 of 134) of late recurrence patients, and 56.0 % (180 of 321) of control patients, while 53.8 % (99 of 184), 51.4 % (69 of 134), and 32.3 % (104 of 321), respectively, received both adjuvant chemotherapy and endocrine therapy. The adjuvant chemotherapy consisted mainly of anthracyclines and/or taxanes.

**Table 1:** Clinicopathological factors according to time of recurrence

				*p* Values		
	Early recurrence (*n* = 184)	Late recurrence (*n* = 134)	No recurrence (*n* = 321)	Early vs no recurrence	Late vs no recurrence	Early vs late recurrence
Age, years						
≤ 50	82 (44.57)	49 (36.57)	120 (37.38)	0.113	0.869	0.151
> 50	102 (55.43)	85 (63.43)	201 (62.62)			
Menopausal status						
Postmenopausal	90 (48.91)	81 (60.45)	171 (53.27)	0.345	0.159	0.041
Premenopausal	94 (51.09)	53 (39.55)	150 (46.73)			
Bilateral breast cancer						
Absent	169 (91.85)	121 (90.30)	319 (99.38)	<0.001	<0.001	0.631
Present	15 (8.15)	13 (9.70)	2 (0.62)			
Tumor size, mm						
≤ 20	49 (26.63)	44 (32.84)	187 (58.26)	<0.001	<0.001	0.23
> 20	135 (73.37)	90 (67.16)	134 (41.74)			
Nodal metastasis						
Negative	93 (50.54)	79 (58.96)	267 (83.18)	<0.001	<0.001	0.136
Positive	91 (49.46)	55 (41.04)	54 (16.82)			
Histological type						
IDC-NST	171 (92.93)	124 (92.54)	297 (92.52)	0.864	0.995	0.892
Others	13 (7.07)	10 (7.46)	24 (7.48)			
Estrogen receptor staining						
< 10 %	16 (8.70)	10 (7.46)	27 (8.41)	0.396	0.367	0.915
10–50 %	57 (30.98)	43 (32.09)	82 (25.55)			
≥ 50 %	111 (60.33)	81 (60.45)	212 (66.04)			
Progesterone receptor staining						
≤ 20 %	85 (46.20)	59 (44.03)	133 (41.43)	0.298	0.609	0.701
> 20 %	99 (53.80)	75 (55.97)	188 (58.57)			
Tumor grade						
1 or 2	131 (71.20)	106 (79.10)	278 (86.60)	<0.001	0.049	0.107
3	53 (28.80)	28 (20.90)	43 (13.40)			
Ki-67 staining						
≤ 20 %	136 (73.91)	114 (85.07)	265 82.55)	0.022	0.507	0.014
> 20 %	48 (26.09)	20 (14.93)	56 (17.45)			
Local recurrence						
Absent	136 (78.61)	96 (71.64)	0			
Present	37 (21.39)	38 (28.36)	0			
Surgical treatment						
Total mastectomy	117 (63.59)	78 (58.21)	106 (33.02)	0.076	<0.001	0.331
Partial mastectomy	67 (36.41)	56 (41.79)	215 (66.98)			
Radiation therapy						
Absent	100 (54.35)	92 (68.66)	149 (46.42)	<0.001	<0.001	0.009
Present	84 (45.65)	42 (31.34)	172 (53.58)			
Adjuvant treatment						
None	13 (7.07)	4 (2.99)	30 (9.35)	<0.001	<0.001	0.021
Chemotherapy only	19 (10.33)	6 (4.48)	7 (2.18)			
Endocrine therapy only	53 (28.80)	55 (41.04)	180 (56.07)			
Combined therapy	99 (53.80)	69 (51.49)	104 (32.40)			
Neoadjuvant chemotherapy						
Absent	168 (91.30)	131 (97.76)	305 (95.31)	0.076	0.198	0.011
Present	16 (8.70)	3 (2.24)	15 (4.69)			
Chemotherapy						
A + T	54 (29.35)	22 (16.42)	42 (13.08)			
A	39 (21.20)	31 (23.13)	40 (12.46)			
T	6 (3.26)	7 (5.22)	13 (4.05)			
CMF	17 (9.24)	12 (8.96)	13 (4.05)			
Other	2 (1.09)	3 (2.24)	3 (0.93)			
None	66 (35.87)	59 (44.03)	210 (65.42)			
Endocrine therapy						
TAM	61 (33.15)	34 (25.37)	68 (21.18)			
TAM + LHRH	30 (16.30)	18 (13.43)	35 (10.90)			
TAM → AI	16 (8.70)	36 (26.87)	72 (22.43)			
TAM + LHRH → AI	1 (0.54)	3 (2.24)	15 (4.67)			
AI	39 (21.20)	27 (20.15)	87 (27.10)			
LHRH	5 (2.72)	6 (4.48)	7 (2.18)			
None	32 (17.39)	10 (7.46)	37 (11.53)			

*Abbreviations: IDC-NST* invasive ductal carcinoma of no special type, *A + T* anthracycline and taxane, *A* anthracycline, *T* taxane, *CMF* cyclophosphamide, methotrexate, and fluorouracil, *TAM* tamoxifen, *TAM + LHRH* tamoxifen and luteinizing hormone-releasing hormone, *TAM → AI* tamoxifen followed by aromatase inhibitor, *TAM + LHRH → AI* tamoxifen and luteinizing hormone-releasing hormone followed by aromatase inhibitor, *AI* aromatase inhibitor, *LHRH* luteinizing hormone-releasing hormone

### Distributions of ALDH1-positive cells

**Fig. 1:**
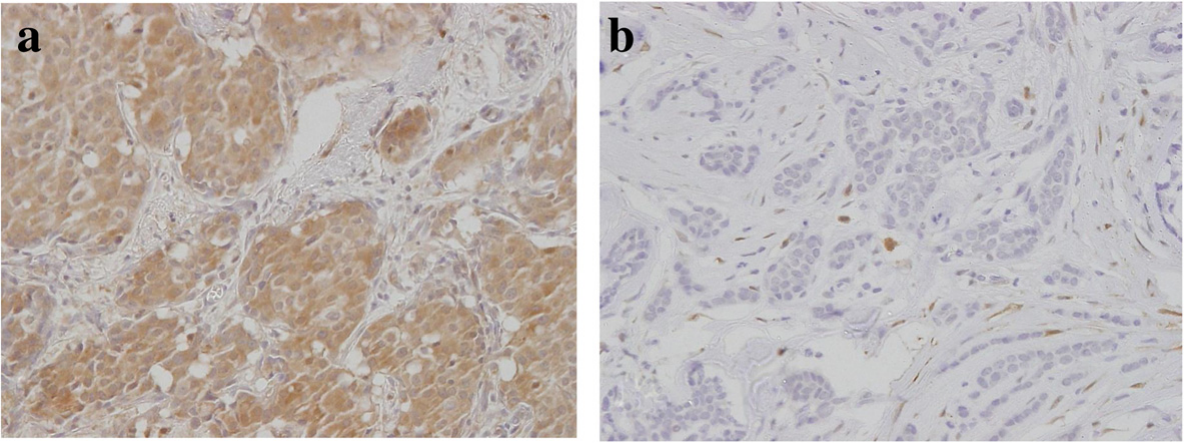
Immunohistochemical staining of aldehyde dehydrogenase 1 (ALDH1) in breast tumor cells. **a** Essentially all of the tumor cells are positive for ALDH1. **b** No ALDH1-positive cells are present

The majority of specimens contained no ALDH1-positive cells, though some showed a patchy epithelial pattern and identifiable hot spots. ALDH1 expression in all or most of the cells in a hot spot was rare. We evaluated the proportion of ALDH1-positive cells in one hot spot, since cancer stem cells represent only a small fraction of the cell population in a tumor. A representative staining pattern in a breast cancer specimen is shown in Fig. 1. The proportions of ALDH1-positive cells were 0–1 % in 560 cases (early recurrence 81.5 % [150 of 184], late recurrence 86.6 % [116 of 134], no recurrence 91.6 % [294 of 321]), 1–5 % in 47 cases (early recurrence 11.4 % [21 of 184], late recurrence 10.4 % [14 of 134], no recurrence 3.7 % [12 of 321]), and 5–100 % in 32 cases (early recurrence 7.0 % [13 of 184], late recurrence 2.9 % [4 of 134], no recurrence 4.6 % [15 of 321]). Only 11 cases showed more than 20 % of tumor cells to be ALDH1-positive (early recurrence 0.6 % [4 of 184], late recurrence 0.3 % [2 of 134], no recurrence 0.7 % [5 of 321]). In many previous studies, 5 % cutoff points were adopted, but there were few ALDH1-positive cases among those with ER-positive/HER2-negative breast cancer. In the present study, cases with ALDH1-positive rates exceeding 5 % were also very few. We regarded positive cases as those in which more than 1 % of cells were ALDH1-positive, because even a small fraction of cancer stem cells might be an important factor. On the basis of our chi-square test employing the 1 % cutoff value, differences among the three groups were statistically significant (*p* = 0.004).

### Associations of ALDH1 expression with clinicopathological factors

We assessed whether ALDH1 expression was associated with clinicopathological factors (Table 2). Both tumor size and nodal metastasis were evaluated clinically. ALDH1 expression in breast cancer specimens correlated significantly with nodal metastasis (*p* = 0.047) and high Ki-67 expression (*p* < 0.001) in the early recurrence group. Age (*p* = 0.077), nodal metastasis (*p* = 0.065), and tumor grade (*p* = 0.058) showed no associations with ALDH1 expression in the late recurrence group. Similarly, no significant differences were detected in the recurrence-free group.

**Table 2:** Associations of aldehyde dehydrogenase 1 expression with clinicopathological factors according to time of recurrence

	Early recurrence			Late recurrence			No recurrence		
	ALDH1^+^	ALDH1^−^	*p* Value	ALDH1^+^	ALDH1^−^	*p* Value	ALDH1^+^	ALDH1^−^	*p* Value
Age, years									
≤ 50	15 (44.12)	67 (44.67)	0.981	10 (55.56)	39 (33.62)	0.077	12 (44.44)	108 (36.73)	0.432
> 50	19 (55.88)	83 (55.33)		8 (44.44)	77 (66.38)		15 (55.56)	186 (63.27)	
Tumor size, mm									
≤ 20	9 (26.47)	40 (26.67)	0.981	4 (22.22)	40 (34.48)	0.288	15 (55.56)	172 (58.50)	0.766
> 20	25 (73.53)	110 (73.33)		14 (77.78)	76 (65.52)		12 (44.44)	122 (41.50)	
Lymph node metastasis									
Negative	12 (35.29)	81 (54.00)	0.047	7 (38.89)	72 (62.07)	0.065	23 (85.19)	244 (82.99)	0.767
Positive	22 (64.71)	69 (46.00)		11 (61.11)	44 (37.93)		4 (14.81)	50 (17.01)	
Estrogen receptor staining									
< 10 %	3 (8.82)	13 (8.67)	0.559	2 (11.11)	8 (6.90)	0.335	3 (11.11)	24 (8.16)	0.831
10–50 %	8 (23.53)	49 (32.67)		8 (44.44)	35 (30.17)		6 (22.22)	76 (25.85)	
≥ 50 %	23 (67.65)	88 (58.67)		8 (44.44)	73 (62.93)		18 (66.67)	194 (65.99)	
Progesterone receptor staining									
≤ 20 %	14 (41.18)	71 (47.33)	0.514	7 (38.89)	52 (44.83)	0.635	13 (48.15)	120 (40.82)	0.461
> 20 %	20 (58.82)	79 (52.67)		11 (61.11)	64 (55.17)		14 (51.85)	174 (59.18)	
Histological grade									
1 or 2	25 (73.53)	106 (70.67)	0.736	11 (61.11)	95 (81.90)	0.058	25 (92.59)	253 (86.05)	0.305
3	9 (26.47)	44 (29.33)		7 (38.89)	21 (18.10)		2 (7.41)	41 (13.95)	
Ki-67 staining									
≤ 20 %	17 (50.00)	119 (79.33)	<0.001	14 (77.78)	100 (86.21)	0.373	20 (74.07)	245 (83.33)	0.247
> 20 %	17 (50.00)	31 (20.67)		4 (22.22)	16 (13.79)		7 (25.93)	49 (16.67)	

*ALDH1* aldehyde dehydrogenase 1

### Comparison of ALDH1 expression in the early and no recurrence groups

ALDH1 expression was significantly higher in the early recurrence group than in the no recurrence group (*p* = 0.001). We selected significant parameters (*p* < 0.20) from among the potentially confounding factors and performed a multivariate analysis in which bilateral breast cancer, age, tumor size, nodal metastasis, tumor grade, and Ki-67 served as categorical variables. Bilateral breast cancer (*p* < 0.001), tumor size (*p* < 0.001), nodal metastasis (*p* < 0.001), tumor grade (*p* = 0.006), and ALDH1 expression (*p* = 0.016) were significant factors predicting early recurrence in the multivariate analysis (Table 3).

**Table 3:** Univariate and multivariate analyses for early recurrence in all cases (early vs no recurrence)

	Early vs no recurrence			
	Univariate analysis	*p* Value	Multivariate analysis	*p* Value
ALDH1, ≤1 %/>1 %	2.468 (1.438–4.274)	0.001	2.140 (1.149–4.003)	0.016
Bilateral breast cancer absent/present	14.156 (3.932–90.478)	<0.001	16.434 (4.111–110.886)	<0.001
Age, ≤50/>50 years	0.742 (0.513–1.073)	0.113	0.748 (0.490–1.142)	0.178
Tumor size ≤20/>20 mm	3.844 (2.604–5.745)	<0.001	2.692 (1.747–4.181)	<0.001
Nodal metastasis negative/positive	4.838 (3.220–7.336)	<0.001	3.728 (2.383–5.876)	<0.001
Tumor grade 1 or 2/3	2.615 (1.666–4.128)	<0.001	2.063 (1.230–3.470)	0.006
Ki-67 staining <20 %/≥20 %	1.670 (1.076–2.586)	0.022	0.855 (0.506–1.427)	0.551

*ALDH1* aldehyde dehydrogenase 1

### Comparison of ALDH1 expression in the late and no recurrence groups

The univariate analysis revealed no significant difference in ALDH1 expression between the late and no recurrence groups (*p* = 0.110). We performed a multivariate analysis in which bilateral breast cancer, menopausal status, tumor size, nodal metastasis, and tumor grade served as categorical variables. In this multivariate analysis, the relationship between ALDH1 expression and late recurrence did not reach statistical significance (*p* = 0.253) (Table 4).

**Table 4:** Univariate and multivariate analyses for late recurrence in all cases (late vs no recurrence)

	Late vs no recurrence			
	Univariate analysis	*p* Value	Multivariate analysis	*p* Value
ALDH1, ≤1 %/>1 %	1.689 (0.883–3.164)	0.110	1.507 (0.738–2.998)	0.253
Bilateral breast cancer absent/present	17.136 (4.645–110.57)	<0.001	18.952 (4.812–126.55)	<0.001
Menopausal status pre/post	0.745 (0.493–1.121)	0.159	0.731 (0.464–1.144)	0.171
Tumor size, ≤20/>20 mm	2.854 (1.878–4.387)	<0.001	2.356 (1.495–3.749)	<0.001
Nodal metastasis negative/positive	3.442 (2.193–5.421)	<0.001	2.940 (1.809–4.790)	<0.001
Tumor grade 1 or 2/3	1.707 (1.001–2.877)	0.046	1.316 (0.727–2.339)	0.358

*ALDH1* aldehyde dehydrogenase 1

### Comparison of ALDH1 expression in the early and late recurrence groups

Employing the chi-square test, the univariate analysis revealed no significant difference in ALDH1 expression between the early and late recurrence groups (*p* = 0.225) (Table 1). An OR was not calculated, because ALDH1 was not selected as a significant parameter (*p* < 0.20).

### Comparison of ALDH1 expression between the early and no recurrence groups receiving chemotherapy and endocrine therapy

We analyzed 203 cases (99 early recurrence, 104 no recurrence) administered both endocrine therapy and chemotherapy to compare clinicopathological factors (Table 6). Tumor size (*p* = 0.004), nodal metastasis (*p* < 0.001), tumor grade (*p* = 0.007), and ALDH1 expression (*p* < 0.001) differed significantly between these early and no recurrence groups by univariate analysis. We selected significant parameters (*p* < 0.20) from among the various conventional confounding factors and performed a multivariate analysis in which age, clinical tumor size, clinical nodal metastasis, tumor grade, and Ki-67 expression served as categorical variables. This multivariate analysis revealed tumor size (*p* = 0.044), nodal metastasis (*p* < 0.001), and ALDH1 expression (*p* < 0.001) to be significant factors predicting early recurrence (Table 5).

**Table 5:** Univariate and multivariate analyses for early recurrence in those receiving endocrine therapy and chemotherapy (early vs no recurrence)

	Early vs no recurrence in combined therapy group			
	Univariate analysis	*p* Value	Multivariate analysis	*p* Value
ALDH1, ≤1 %/>1 %	4.054 (1.797–10.080)	<0.001	4.625 (1.881–12.474)	<0.001
Age, ≤50/>50 years	0.612 (0.349–1.067)	0.083	0.623 (0.330–1.164)	0.138
Tumor size, ≤20/>20 mm	2.553 (1.336–5.036)	0.004	2.130 (1.018–4.601)	0.044
Nodal metastasis negative/positive	4.357 (2.430–7.979)	<0.001	3.856 (2.026–7.519)	<0.001
Progesterone receptor staining, <20 %/≥20 %	1.035 (0.593–1.807)	0.903	Not selected	
Ki-67 staining, <20 %/≥20 %	1.592 (0.858–2.983)	0.140	0.791 (0.377–1.634)	0.529
Tumor grade 1 or 2/3	2.625 (1.285–5.595)	0.007	2.098 (0.940–4.848)	0.070

*ALDH1* aldehyde dehydrogenase 1

### Comparison of ALDH1 expression between the early and no recurrence groups receiving endocrine therapy alone

We analyzed 233 (early recurrence *n* = 53, no recurrence 180) patients given endocrine therapy alone to compare clinicopathological factors between these cases. Tumor size (*p* < 0.001), lymph node metastasis (*p* = 0.002), and tumor grade (*p* < 0.001) were significant in the univariate analysis in which we compared the early recurrence and no recurrence groups. ALDH1 expression was not a predictor of early recurrence in these patients receiving endocrine therapy alone (*p* = 0.224) (Table 6).

**Table 6:** Univariate and multivariate analyses for early recurrence in those receiving endocrine therapy alone (early vs no recurrence)

	Early vs no recurrence in endocrine therapy group			
	Univariate analysis	*p* Value	Multivariate analysis	*p* Value
ALDH1, ≤1 %/>1 %	1.804 (0.651–4.609)	0.244	Not selected	
Age, ≤50/>50 years	0.996 (0.526–1.932)	0.992	Not selected	
Tumor size, ≤20/>20 mm	6.182 (3.208–12.383)	<0.001	4.910 (2.469–10.076)	<0.001
Nodal metastasis negative/positive	3.344 (1.543–7.181)	0.002	2.894 (1.229–6.777)	0.002
Progesterone receptor staining, <20 %/≥20 %	0.708 (0.382–1.313)	0.272	Not selected	
Ki-67 staining, <20 %/≥20 %	1.441 (0.619–3.159)	0.383	Not selected	
Tumor grade 1 or 2/3	2.615 (1.666–4.128)	<0.001	2.820 (1.296–6.111)	<0.001

*ALDH1* aldehyde dehydrogenase 1

### Survival after distant recurrence

**Fig. 2:**
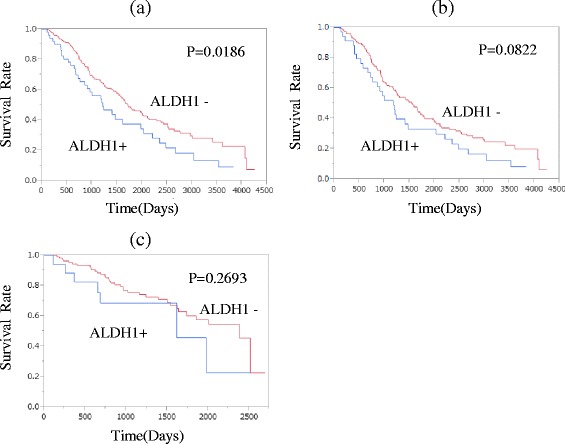
Survival time from recurrence detection until death due to breast cancer. *ALDH1* aldehyde dehydrogenase 1. **a** all cases with recurrence. **b** early recurrence cases. **c** late recurrence cases

We analyzed 318 cases (early recurrence *n* = 184 [58 %], late recurrence 134 [42 %]) to compare overall survival from the time of recurrence detection between ALDH1-positive and ALDH1-negative cases (Fig. 2). The median follow-up duration from the detection of recurrence until death due to breast cancer was 39 (0–141) months in the early recurrence group and 34 (0–89) months in the late recurrence group. The Kaplan-Meier method showed a significant difference between ALDH1-positive and ALDH1-negative cases (*p* = 0.019). Moreover, the Kaplan-Meier method revealed a trend for higher ALDH1 expression in the early recurrence group (*p* = 0.082), while there was no difference in the late recurrence group (*p* = 0.27). Univariate analysis of all cases with recurrence revealed ALDH1 expression, nodal metastasis, and tumor grade to be significant prognostic factors. We selected significant parameters (*p* < 0.20) from among various conventional confounding factors and performed a multivariate analysis in which nodal metastasis, PgR, tumor grade, and Ki-67 expression served as categorical variables. In this multivariate analysis, lymph node metastasis (*p* = 0.036) and tumor grade (*p* = 0.038) were found to be independent prognostic factors, while ALDH1 expression was not (*p* = 0.059) (Table 7).

**Table 7:** Univariate and multivariate analyses for survival time from recurrence detection until death due to breast cancer

	Survival time			
	Univariate analysis	*p* Value	Multivariate analysis	*p* Value
ALDH1, 1 %/>1 %	1.552 (1.057–2.218)	0.025	1.451 (0.985–2.085)	0.059
Age, ≤50/>50 years	1.160 (0.856–1.581)	0.338	Not selected	
Bilateral breast cancer absent/present	0.813 (0.438–1.376)	0.438	Not selected	
Tumor size, ≤20/>20 mm	1.062 (0.764–1.503)	0.721	Not selected	
Nodal status negative/positive	1.500 (1.109–2.032)	0.008	1.387 (1.020–1.890)	0.036
Progesterone receptor staining, <20 %/≥20 %	0.789 (0.582–1.072)	0.130	0.778 (0.572–1.060)	0.111
Ki-67 staining, <20 %/≥20 %	1.270 (0.888–1.781)	0.184	1.208 (0.839–1.706)	0.301
Tumor grade 1 or 2/3	1.519 (1.080–2.105)	0.016	1.438 (1.019–1.999)	0.038

*ALDH1* aldehyde dehydrogenase 1

## Discussion

Our results provide important insight into the chemoresistant nature of cancer stem cells. Furthermore, intensive chemotherapy might alter the significance of the ALDH1 marker in clinical settings. Although several previous studies have suggested an association between ALDH1 and clinical outcomes in breast cancer, our analyses showed much higher ALDH1 expression in early recurrence cases of patients receiving both endocrine therapy and chemotherapy, as compared with recurrence-free patients. Furthermore, ALDH1 was associated with an aggressive phenotype in the early recurrence group. We speculate that ALDH1 has the capacity to induce chemoresistance of highly proliferative breast cancer cells, which might explain why we identified several early recurrence cases among those patients who had received adjuvant chemotherapy for ER-positive/HER2-negative breast tumors.

The reported percentages of ALDH1-positive cases range from 7.0 % to 59 % [2, 6, 13, 14, 16–26]. This broad range may reflect differences in cutoff points, sampling methods, and study populations among studies. Ricardo et al. reported ALDH1 expression rates in different breast cancer subtypes [27]. The rates were 5.1 % in the luminal A, 12.2 % in the luminal B, and 25 % in the basal types, while the rate was 12.29 % in the HER2 type. In the present study, the rates of ALDH1 positivity at a 1 % cutoff value were 18.4 %, 13.4 %, and 8.4 % in patients with early, late, and no recurrence, respectively, among those with ER-positive/HER2-negative breast cancer. We found a significant difference in ALDH1 expression between the early recurrence patients, at the time of recurrence, and those who remained recurrence-free. We also investigated the time from the detection of recurrence until death due to breast cancer according to ALDH1 expression. Univariate, but not multivariate, analysis showed patients with ALDH1-positive breast cancer to have a shorter survival time. This observation suggests that the presence of ALDH1-positive cancer stem cells correlates with early recurrence and shorter survival. Researchers in another study found patients with ALDH1-positive tumors to have poorer outcomes than those with ALDH1-negative tumors [6, 20, 26, 28–31]. However, the authors of other reports noted no association of ALDH1 expression with poor outcomes [13, 21, 32, 33].

The differences among study results may be attributable to differences in sample sizes, follow-up periods, tissue microarray use, and use of various cutoff values for ALDH1 staining. Yoshioka et al. highlighted the importance of long-term follow-up, of employing a low cutoff value, and of not using tissue microarrays for evaluating ALDH1 expression [29]. In the present study, we examined the data of 639 patients, many of whom were observed for at least 10 years. We used an ALDH1 cutoff value of 1 %, which was lower than cutoffs employed in most other studies. We used immunohistochemically stained sections, examined whole sections, and evaluated one hot spot in each section. In a previous report, Tsang et al. reported ALDH1 alone not to be an independent prognostic factor for luminal (ER-positive, HER2-positive or HER2-negative) breast cancers [34]. However, they used tissue microarray slides for IHC staining and used an ALDH1 cutoff value of 5 %. Tissue microarray slides might be of limited utility for detecting minor populations of cancer stem cells. To identify such populations, we screened whole sections and evaluated a cluster of cancer stem cells in one hot spot. These methods might be optimal for identifying patient populations with a poor prognosis. Further study is needed to identify the optimal means of determining how cancer stem cells impact prognosis. It is necessary to identify populations of cancer stem cells potentially associated with a poor prognosis.

In a previous study, the effect of ALDH1 on clinical outcomes was found to be stronger in a lymph node metastasis-positive subgroup and a neoadjuvant chemotherapy setting [2, 14, 19, 23, 29, 30, 35]. In an in vitro study, cancer stem cells showed chemoresistance traits [7, 35]. Large tumor burden, locally advanced tumor, epithelial-mesenchymal transition, high cellular proliferation, and adjuvant and neoadjuvant settings might augment the role of cancer stem cells in breast cancer recurrence. Therefore, we analyzed these data in a subgroup receiving both endocrine therapy and chemotherapy in the adjuvant setting. These subgroup analyses showed higher ALDH1 expression in early recurrence patients receiving both endocrine therapy and chemotherapy, as compared with recurrence-free patients. However, there was no significant difference in those receiving endocrine therapy alone. These findings highlight ALDH1 as a detoxifying enzyme, suggesting that ALDH1-positive cells may contribute to the development of resistance to adjuvant chemotherapeutic agents.

Finally, the relationships between ALDH1 protein expression and clinicopathological factors influencing ER-positive/HER2-negative breast cancers were investigated. In a previous study, ALDH1-positive tumors were found to have aggressive phenotypes, such as triple-negative, HER2-positive, high histological grade, high Ki-67 expression, and advanced TNM stage [13, 17, 18, 27, 28, 34, 36]. We found ALDH1-positive tumors to be significantly associated with high Ki-67 and lymph node metastasis in cases with early recurrence. Morimoto et al. reported that cells staining for MIB-1 did not correspond to those staining for ALDH1 [13]. One of the most important breast cancer markers is Ki-67, which predicts poor outcomes and identifies which patients would benefit from adjuvant chemotherapy. Combining the features of cancer stem cells and high proliferative activity might allow the identification of a group prone to early recurrence. However, according to a report by Liu et al., microarray analysis revealed ALDH1-positive cells to express higher levels of Ki-67 than ALDH1-negative cells [12]. ALDH1-positive breast cancer stem cells are associated with a proliferative state. These epithelial proliferative traits might contribute to breast cancer recurrence. Alternatively, quiescent ALDH1-positive tumor cells might be less likely to proliferate. Furthermore, Tsang et al. reported that basal marker expression can enhance the prognostic value of cancer stem cells in luminal (ER-positive, HER2-positive or HER2-negative) breast cancers [34]. Importantly, breast cancer is a heterogeneous disease. Probably, with regard to breast cancer traits, the significance of ALDH1-positive cancer cells in terms of contributing to prognosis also varies among tumor types. It is necessary to understand the heterogeneity of cancer stem cells (i.e., which cell types can form metastatic nodules and which cannot) to identify subgroups with metastatic potential and a poorer prognosis.

The major limitation of the present study is its retrospective design. Also, the patients were treated with various forms of adjuvant therapy that might have affected both recurrences and outcomes. Therefore, additional prospective studies with well-planned cohorts are needed to confirm our findings.

## Conclusions

We identified more ALDH1-positive cases among patients with early recurrence of ER-positive/HER2-negative breast cancer than among those who remained recurrence-free for at least 10 years. In these groups, ALDH1-positive cases had an aggressive phenotype. Patients with ALDH1-positive breast cancer also tended to have a shorter survival time. Further study is needed to understand the heterogeneity of cancer stem cells and to confirm their role in the development of resistance to chemotherapy.

## Abbreviations

A, anthracycline; AI, aromatase inhibitor; ALDH1, aldehyde dehydrogenase 1; A + T, anthracycline and taxane; CMF, cyclophosphamide, methotrexate, and fluorouracil; DAB, 3,3′-diaminobenzidine; ER, estrogen receptor; HER2, human epidermal growth factor receptor 2; IDC-NST, invasive ductal carcinoma of no special type; IHC, immunohistochemical; IRB, institutional review board; ISH, in situ hybridization; LHRH, luteinizing hormone-releasing hormone; NHO, National Health Organization; PgR, progesterone receptor; T, taxane; TAM, tamoxifen; TAM → AI, tamoxifen followed by aromatase inhibitor; TAM + LHRH, tamoxifen and luteinizing hormone-releasing hormone; TAM + LHRH → AI, tamoxifen and luteinizing hormone-releasing hormone followed by aromatase inhibitor
